# Barriers and facilitators to the participation and engagement of primary care in shared-care arrangements with community mental health services for preventive care of people with serious mental illness: a scoping review

**DOI:** 10.1186/s12913-023-09918-2

**Published:** 2023-09-11

**Authors:** Sharon M. Parker, Katrina Paine, Catherine Spooner, Mark Harris

**Affiliations:** 1 Centre for Primary Health Care and Equity, University of New South Wales, Sydney, New South Wales Australia; 2 Susan Wakil School of Nursing, Faculty of Medicine and Health, University of Sydney, Sydney, New South Wales Australia

**Keywords:** Serious or severe illness, Shared care, Preventive care, Primary care, General practice

## Abstract

**Background:**

People with serious mental illness die about 20 years earlier than the general population from preventable diseases. Shared-care arrangements between general practitioners and mental health services can improve consumers’ access to preventive care, but implementing shared care is challenging. This scoping review sought to describe current evidence on the barriers and facilitators to the participation and engagement of primary care (specifically general practitioners) in shared-care arrangements with community mental health services for preventive health care of this population.

**Methods:**

We searched Medline, Embase, CINAHL, Scopus, APA PsychINFO and EBM Reviews from 2010 to 2022. Data was extracted against a Microsoft Excel template developed for the review. Data was synthesised through tabulation and narrative methods.

**Results:**

We identified 295 records. After eligibility screening and full-text review, seven studies were included. Facilitators of engagement included a good fit with organisation and practice and opportunities to increase collaboration, specific roles to promote communication and coordination and help patients to navigate appointments, multidisciplinary teams and teamwork, and access to shared medical/health records. Barriers included a lack of willingness and motivation on the part of providers and low levels of confidence with tasks, lack of physical structures to produce capacity, poor alignment of funding/incentives, inability to share patient information and challenges engaging people with severe mental illness in the service and with their care.

**Conclusion:**

Our results were consistent with other research on shared care and suggests that the broader literature is likely to be applicable to the context of general practitioner/mental health services shared care. Specific challenges relating to this cohort present difficulties for recruitment and retention in shared care programs. Sharing “goals and knowledge, mutual respect” and engaging in “frequent, timely, accurate, problem-solving communication”, supported by structures such as shared information systems are likely to engage primary care in shared care arrangements more than the traditional focus on incentives, education, and guidelines.

**Supplementary Information:**

The online version contains supplementary material available at 10.1186/s12913-023-09918-2.

## Background

People with serious/severe mental illness (PWSMI) have a 13-to-30-year shorter life expectancy than the general population [1–3]. A number of factors contribute to this including higher rates of modifiable cardiometabolic risk factors such as smoking, hyperlipidaemia, hypertension, obesity, diet, and sedentary lifestyle [4, 5]. Use of psychotropic medications are also associated with increased risk of obesity, dyslipidaemia, Type 2 diabetes and cardiovascular risk [6]. Subsequently, this cohort is more likely to die of cardiovascular disease and respiratory disease than the general public [7]. Around 80% of PWSMI are diagnosed with at least one chronic physical health condition, and around 55% endure at least two [4]. The true number may in fact be higher due to low rates of routine screening and detection in these individuals [5, 8].

Poor uptake of preventive and proactive use of primary care is related to a number of health system issues (e.g., stigma, poor communication), consumer issues (e.g., cognitive difficulties, social circumstances and low health literacy) and social determinants of health [9, 10]. Nonpsychiatric inpatient admissions, length of stay, hospital readmission rates, and emergency department visits have been shown to be higher in medical patients with severe/serious mental illness (SMI) compared to patients without SMI [6]. Within Australia, complex funding arrangements mean primary care and mental health services are administered and funded separately, and this separation adds to the challenges of providing quality physical health care to PWSMI [11]. Primary care is funded through the Australian government and a universal public insurance scheme (Medicare) [12]. Responsibility for funding and regulating mental health services in Australia is shared among the Australian, state and territory governments, however the state and territory governments fund and manage community mental health services [13].

Over recent years, various models of care have been developed that aim to improve the way primary care and mental health services work together [14]. These models have largely been used to improve the management of mental health conditions (particularly depression) within primary care. Many different terms are used to describe these collaborative activities [15]. Integrated care is a broad term that refers to the provision of health services to meet the needs of individuals in a coordinated way [16]. Such care may be organised in a combination of ways ranging from simple linkage, through to coordination and the full integration of services [17]. Shared care is one system for achieving integration [18]. It refers to the planned delivery of care with enhanced information exchange [19] where both parties maintain ongoing involvement in patient care, share information and clinical responsibilities and proactively agree on common processes [20].

Systematic reviews of shared and collaborative arrangements between primary care and specialist services (including mental health) have shown mixed results for patients with chronic disease such as hypertension, kidney disease, stroke, depression, and anxiety [18, 21]. Shared or collaborative arrangements for those with SMI is less researched and tends to focus predominantly on how these models affect mental health management and outcomes [18]. A notable gap in the evidence includes how mental health services and primary care can better collaborate to create systems of care in which PWSMI are regularly seen by a General Practitioner (GP) while receiving specialist mental health care, and how mental health and primary care services can improve communication and integration of services [22].

This scoping review aimed to inform the work being conducted in Sydney Local Health District through the Shared Health Arrangements Research & Development (SHAReD) trial (https://www.unsw.edu.au/research/cphce/research/projects/shared--shared-health-arrangements-research---development) by identifying the barriers and facilitators to the participation and engagement of primary care (specifically GPs) in shared-care arrangements with community mental health services for preventive care of PWSMI.

## Methods

The protocol for this review was published in Open Science Framework [23].

### Research question and definitions

The research question addressed by this scoping review is: What are the barriers and facilitators to the participation and engagement of primary care in shared-care arrangements with community mental health services for preventive care of PWSMI?

This review adopted the following definitions:

- ***Shared care:*** “A structured system for achieving integration of care across multiple autonomous providers and services with both primary and secondary care practitioners contributing to elements of a patient's overall package of care. Shared care involves some agreement about the shared activities and levels of responsibility for each provider and appropriate communication processes to support this integration. A shared-care arrangement may involve any combination of government, non-government or private sector providers” [18].
- ***Serious mental illness/Severe mental illness:*** The terms serious or severe mental illness (SMI) are often used interchangeably [24] and refers to mental illness “which is severe in degree and persistent in duration, that causes a substantially diminished level of functioning in the primary aspects of daily living” [25]. SMI includes (but is not limited to) psychotic illnesses (primarily schizophrenia or bipolar affective disorder), severe depression, and severe anxiety disorders. It affects about 3% of Australians [26, 27].
- ***Preventive care:*** Preventive health care is a range of activities with the goal to reduce the risk of ill-health or disability within an identified population [28].

### Search strategy

Currently there is no Medical Subject Heading (MeSH) specifically for ‘shared care’, and the term is frequently used interchangeably with other terms such as ‘collaborative care’ and ‘integrated care’. Through testing and review of literature in the field, we identified several MeSH terms and additional key words for each of the major concepts within the research question (Table 1). The search strategy was refined within Medline prior to it being adapted for use in each database searched. A university librarian reviewed the search strategies prior to their application.

**Table 1:** Search strategy

Concept	Terms Used
Shared care	"Cooperative Behavior"[Mesh] OR "Electronic Health Records"[Mesh] OR "Health Information Exchange"[Mesh] OR "Delivery of Health Care, Integrated"[Mesh] OR "Delivery of Health Care/organization and administration"[Mesh] OR "Interprofessional Relations"[Mesh] OR "Interdisciplinary Communication"[Mesh] OR "Case Management"[Mesh] OR "Case Management/organization and administration"[Mesh] OR "Patient Care Planning/organization and administration"[Mesh] OR "Attitude of Health Personnel"[Mesh] OR “shared care plan”[TW] OR “shared care “[TW] OR “shared care”[TIAB] OR “collaborative care”[TW] OR “coordinated care”[TW]
Primary Care	"Primary Health Care"[Mesh] OR "Family Practice"[Mesh] OR "General Practice"[Mesh]
Community mental health	"Community Mental Health Services"[Mesh] OR "Community Mental Health Centers"[Mesh] OR "Mental Health Services"[Mesh]
Serious Mental illness	"Mentally Ill Persons"[Mesh] OR "Mental Disorders"[Mesh] OR "Schizophrenia Spectrum and Other Psychotic Disorders"[Mesh] OR "Bipolar Disorder"[Mesh] OR "Psychotic Disorders"[Mesh] OR “serious mental illness” [TW] OR “severe mental illness”[TW] OR “serious psychiatric illness”[TW]
Preventive care	"Preventive Health Services"[Mesh] OR "Diagnostic Services"[Mesh] OR "Primary Prevention"[Mesh] OR "Secondary Prevention"[Mesh] OR "Hypertension"[Mesh] OR "Diabetes Mellitus"[Mesh] OR "Hyperlipidemias"[Mesh] OR "Comorbidity"[Mesh] OR "Chronic Disease"[Mesh] OR "Multiple Chronic Conditions"[Mesh] OR "Disease Management"[Mesh] OR "Obesity Management"[Mesh] OR “physical health care”[TW] OR “physical health”[TW] OR “disease screening”[TW] OR “cancer screening”[TW]

A search log documented the name of each database, the number of articles retrieved, the date of coverage and date searched (Table 2). Each search was stored within the platform so it could be re-run. Search results were imported to the Endnote reference manager and duplicate citations were removed and stored in an Endnote duplicates library.

**Table 2:** Databases searched

Database	Platform	Date of search
Medline	Ovid	27/8/2021
Embase	Ovid	27/8/2021
EBM Reviews	Ovid	27/8/2021
CINAHL	EBSCO	6/9/2021
Scopus	Elsevier	6/9/2021
PsychInfo	Ovid	7/10/2021

Table 3 outlines the inclusion and exclusion criteria applied. Included were published data from the last 10 years (2011–2021) regardless of study design. Ten years was chosen as a suitable timeframe as it coincided with substantial Australian mental health reform[29–31], the introduction of shared care between mental health and primary care services for the management of consumers on clozapine [32, 33] and the establishment of shared care models in aspects of healthcare where non-GP specialists and GPs need to work together such as antenatal shared care, cancer, and diabetes [20]. Ten years was therefore seen as timely in which to find relevant data that we could incorporate into the (SHAReD) trial. Inclusion was limited to publications in English and those originating within Organisation for Economic Cooperation and Development (OECD) countries (as we wanted comparative health systems to that of Australia). Excluded were editorials, letters, opinion pieces, and protocols for trials, as well as studies of children and adolescents.

**Table 3:** Inclusion and exclusion criteria

Domain	Inclusion	Exclusion
Setting	Family/General practice -First/generalised port of call for medical services in the community (metropolitan, rural, regional). These include small, single GP practices or larger scale practices, with multiple GPs. Corporate run practices and GP super clinics were included but may be separated in the analyses as their structure may impact the generalizability of research produced in this setting (e.g., funding, resourcing). This includes primary care services provided by a nurse practitioner Mental health services community based mental health services characterised by multidisciplinary teams providing care through monitoring and case management (short and long term) and coordination of care for patients experiencing SMI (according to the applied definition)	Primary care based in hospitals if they are NOT an independent service (i.e., part of the mental health service) PC Nurse practitioner if they are not part of a PC or mental health team Secondary and tertiary mental health services: - Private psychiatrists - In-patient units - Hospital-based outreach programs
Participants	Adults (18 +) with SMI (as defined) Dual diagnoses of SMI and substance abuse disorders provided the shared-care arrangement is related to physical care and not the management of substance addiction/abuse	Those aged below 18 years of age unless the adult population is analysed separately Predominant diagnosis of intellectual disability Patients with mental health conditions that do not meet the enduring and persistent nature of SMI (i.e., acute depression)
Concept/intervention	The shared-care arrangement must be a system put in place to promote the physical/preventive health (as defined) of people with SMI (as defined) and be between primary care and mental health services (as defined)	Interventions that do not meet the study definition for shared care Shared-care arrangements that are not focused on physical or preventive care for people with SMI The management of mental health conditions within primary care (e.g., depression/anxiety) Cancer management for PWSMI Specialised models of care that promote integration such as co-location and care navigators
Outcomes	Barriers to and facilitators of engagement by GPs, PC services, and mental health services This could include but was not limited to: Communication methods/triggers for communication Systems to promote communication/information sharing systems/electronic systems Templates, forms, on-line tools Formal agreements Service structure/models/pathways Funding/ costs Training/education Shared expertise Access to appropriate services Health delivery outcomes Personal factors Organisational factors/ changes to service delivery	Patient outcomes including health indicators (HBA1C, BP, cholesterol etc.), mental health indicators (e.g., depression scores), satisfaction with Shared-care arrangements, self-management measures, QoL etc Shared decision making between providers and patients Carer outcomes such as satisfaction with shared-care arrangements
Country of Origin/language	Publications in English produced in OECD countries (https://www.oecd.org/about/document/ratification-oecd-convention.htm)	All other countries and languages
Publication type	Research papers using any quantitative and qualitative methodology Systematic reviews and evidence reviews provided they focused on ‘shared care’ according to our definition, within our specified settings, and our target group	Editorials, letters, commentary and opinion pieces, protocols for studies, conference abstracts, vignettes
Years of publication	2010 to current	Publications outside of this timeframe

### Study selection

Studies were excluded if a good proportion (75% or more) of the study sample did not have a mental health condition consistent with the persistent and enduring nature of SMI. We also excluded studies that did not fully describe their population, or those that used vague or general ways to define their population (e.g., those with ‘mental health issues’ or ‘behavioural health issues’).

Because of our interest in the interaction between primary care and mental health services that are typically separate entities within the Australian health care system, we excluded studies where the primary care service and the mental health service did not operate independently of each other; that is, where the two services were already combined in some way (e.g., shared systems, shared staff, shared funding). Services could be located close to each other if there was evidence from the publication of independence. We also excluded studies in which the interaction was between primary care and a service that did not align with our experience of a public community mental health service (i.e., a service providing assessment and support to people with mental illness living in the community). This excluded hospital based, outpatient or outreach services, and specialist services such as private psychiatrists or mental health in-patient units.

To be included in our review, the interaction described in the study needed to be specifically focused on the prevention or management of a physical health condition for PWSMI. We excluded studies of cancer care due to the specialised nature of this management.

Two authors (SP and KP) reviewed the titles and abstracts of all citations identified from the database search. Full-text articles were retrieved where it was not possible to determine inclusion/exclusion requirements from the title and abstract alone. Where there was disagreement or uncertainty, this was resolved through discussion or a third assessment (MH or CS). Reference lists of the most relevant citations were reviewed for additional literature that might have been missed through searching. We contacted the author of one publication where the final outcomes publication could not be sourced.

### Data extraction and reporting

A data extraction template was developed in Excel. Two authors (SP and KP) independently extracted data and collaborated to finalise the extraction of included studies. A narrative synthesis with tabular mapping was used to describe and summarise the data identified through the database search. As this review was primarily interested in identifying the barriers to and facilitators of the engagement of primary care to shared-care arrangements, all reported barriers and facilitators were extracted from the included studies and organised under specific headings for the results.

The Preferred Reporting Items for Systematic reviews and Meta-Analyses extension for Scoping Reviews (PRISMA-ScR, 2018) provided as a Supplementary file was used to guide reporting (1) [34]. The checklist contains 20 essential reporting items and two optional items (http://www.prisma-statement.org/Extensions/ScopingReviews).

## Results

### Study selection

**Fig. 1:**
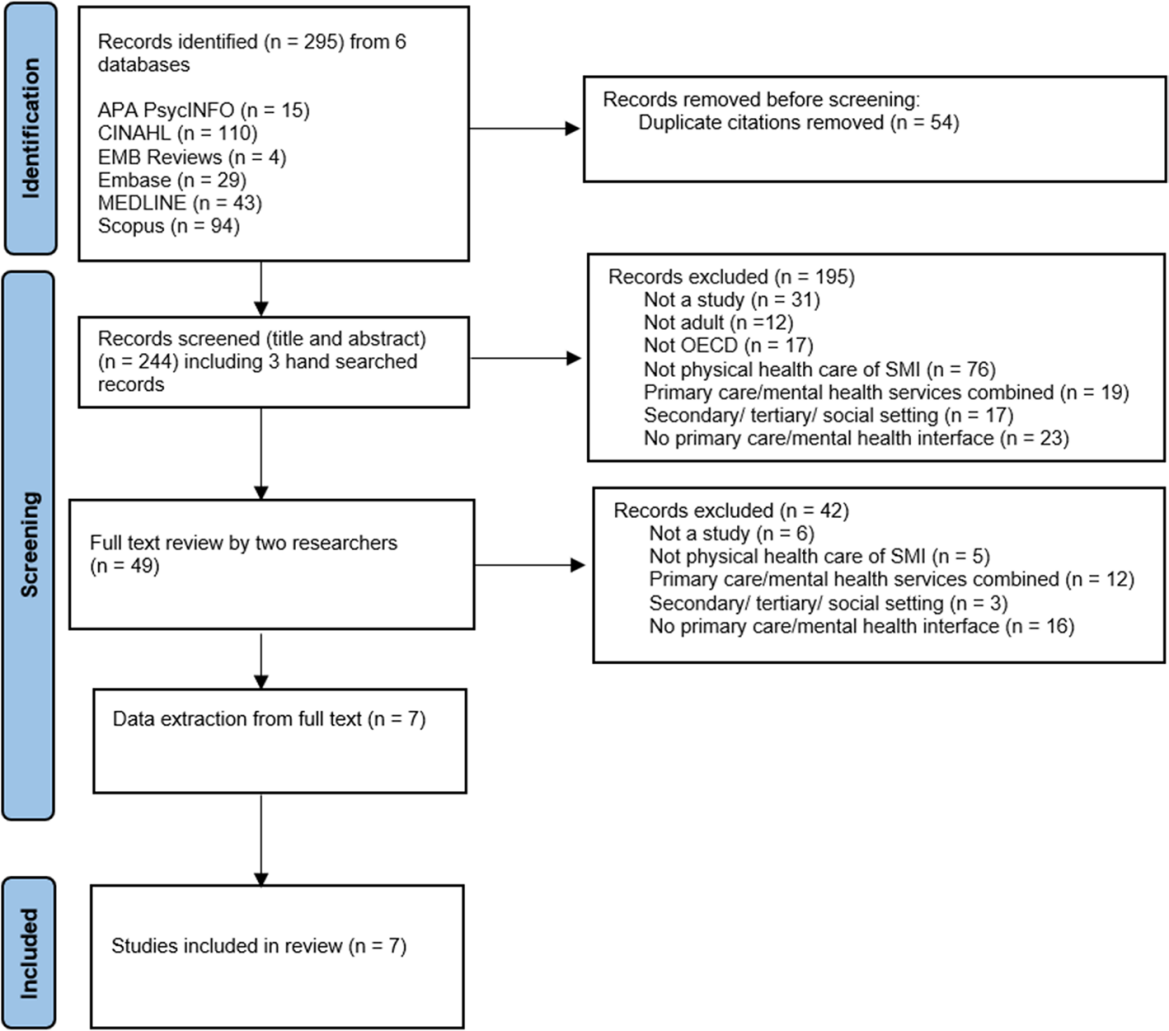
PRISMA 2020 Flow Diagram

As depicted in the Prisma flow diagram [35] (Fig. 1), our search identified 295 citations from 6 bibliographic databases, with 54 duplicates subsequently removed. We screened 244 citations using titles and abstracts which included these citations plus three additional citations obtained through handsearching. A further 195 citations were excluded at this point. We retrieved 49 full text articles of which 42 were excluded, leaving 7 included studies.

### Description of the included studies

The seven included studies (Table 4) originated from Australia, the United Kingdom (UK), and the United States (US). A narrative synthesis was used to describe the studies and to summarise the barriers and facilitators identified within the studies.

**Table 4:** Description of the included studies

Author/Year	Country	Study type	Aim	Population and setting	Intervention description	Outcome: physical health,	Outcome: facilitators	Outcome: barriers
Fitzpatrick 2017 [36]	Australia	Case study approach using a conceptual framework	To examine the integration of primary and community-based specialist care for people with SPMI in a small rural town in Australia to better understand the dynamics of integrated care	People living with severe and persistent mental illnesses (SPMI) Single rural general practice The GP clinic was located in an adjacent building to the Community Mental Health Service (CMHS) service. The CMHS service was co-located with other services including a diabetic educator, women’s health and drug and alcohol services	Collaborative monthly clinic for SPMI clients conducted at a single rural general practice. Appointments were arranged by a member of the CMHT who also provided transport for their client. Results of the consultations were documented in the GP and CMHS records. Consultations were paid through Medicare	Not reported	1. GP who was committed to staying in the town and happy to volunteer his clinic 2. Improvement in skills and knowledge was a benefit achieved from working with the CMHS and people with SPMI	1. Lack of GP confidence to care for clients with complex SPMI. This, reinforced by their understanding of normal professional practice and a poor alignment of incentives and payments with patient needs were seen as reinforcing established practice, effectively excluding people with SPMI 2. GP Clinic influenced by the political and economic understandings that determined what should be considered as cost-effective and efficient practice (e.g. multiple people in a consultation as time wasting/inefficient) 3. Limited capacity of the clinic to collaborate with other community and social services to provide recovery-oriented mental health practice. Despite incorporating key aspects of recovery-oriented practice such as advocacy, social inclusion, and the support of individuals and their families to self-manage their physical and mental health, the study found less evidence for the necessary infrastructure and supportive relationships required to strengthen the recovery orientation of service delivery to include housing, employment, and income and vocational training support – elements that are key to achieving recovery outcomes. 4. Lack of available fees for missed appointments
Hunt 2016 [37]	UK	Mixed methods process evaluation	To understand the function of the Community and Physical Health Co-ordinator (CPHC) role as a ‘boundary spanner’ between primary and community care, with the aim of improving collaboration of mental and physical health care for service users with (SMI)	Severe mental illness (SMI) -schizophrenia and bipolar disorder/ schizophrenia and psychosis Five general practices (GPs) and one Community Mental Health Team (CMHT)	Community and Physical Health Co-ordinator (CPHC) role to ‘span the boundaries’ between primary care and community mental health services. Two CPHC’S were existing staff of the CMHT and worked between mental health and primary care The purpose of the role was to improve communication and collaboration via a regular multidisciplinary team (MDT) meeting. An action plan was developed for each client and monitored through the MDT meeting using a traffic light system (green, amber, red) which indicated the individuals responsible for the action. This was communicated to relevant professionals across the services. MDT meetings occurred at the general practice and were structured based on the size of each practice.	Not reported. The study reports that outcomes from the multidisciplinary team meetings and audit of GP systems included disease reviews and actions related to physical health checks and medication reviews. The study reports improvement in recording of CV risk and an improvement in the information recorded. Recording of all four risk factors (systolic BP, HDL, smoking status and BMI) improved from 38% of service users (n = 115) to 58% (n = 79) post project.	1. CPHC role - Split function was crucial for embedding the CPHC role in practice as it allowed CPHCs to retain their care coordination skills, retain access to appropriate service user and organisational information, meetings and discussions and importantly helped to maintain trust and respect from peers. - Being embedded in the team was an important aspect of the role for maintaining trust and accessing relevant service user information, both of which were crucial to the successful implementation of the role - Protected time was a key factor for success and enabled the CPHCs to be self-organising. They were responsible for liaising with teams from primary and community care to design a method of working which was appropriate, relevant and locally tailored to the needs of the service. 2. Multidisciplinary team meetings—Traffic light system which allocated tasks to specific individuals and monitored when these tasks were completed as well as highlighting unfinished tasks. The meetings were a key facilitator for designing and delivering appropriate physical health care management plans. It provided an avenue for new learnings and opportunities for collaboration.	1. Information in the paper-based care plan was frequently out of date and insufficient. 2. The CPHC had improved care for some users but hard to reach groups were difficult to access.
Nover 2014 [38]	USA	CalMEND Pilot Collaborative Descriptive—Plan-Do-Study-Act (PDSA) model was used to evaluate changes to clinic structure, to describe implementation and track organisational changes made through the pilot	To describe the process of implementing and evaluating a specific health–mental health integration program at Placer County Community Clinic (PCCC) so it can be used to assist other primary care clinics in the process of integrating primary care and behavioural or mental health systems	Individuals with Serious mental illness (SMI) defined as major depressive disorder (recurrent), bipolar disorder, and*/*or schizophrenic disorders who also had a diagnosis of, or risk factors for, hypertension, coronary artery disease, dyslipidaemia, and*/*or diabetes. Rural primary care clinic/rural health centre (PCCC) serving primarily low-income individuals. PCCC had multiple primary care providers and one psychiatrist co-located on site. Partnership model with a mental health agency (Placer County Adult System of Care (ASOC). This was located at the same site as the clinic but operated separately.	Pilot CPCI service: Expanded services for patients with mental illness through staffing and service delivery changes at the PC clinic. ASOC employed a social worker to coordinate the program including case management, organisational development, counselling, and motivational and educational components for clinic patients. Clinic administrators selected a registered nurse who was already employed at the PC clinic to work part time with the CPCI program at the clinic to conduct medical assessments and teaching. The clinic identified goals which were documented on a treatment plan and used to guide the patient’s future treatment.	Not reported	1. Nurse led care coordination for medication support, illness management and referral/follow up was the most frequently requested service. 2. Assessment appointments scheduled proximally to other clinic appointments decreased inconvenience for patients who often had inconsistent transportation options and limited ability to return to the clinic repeatedly. 3. Psychiatric and primary care providers worked closer together to improve patient health outcomes and all providers were educated about the importance of treating mental and physical health simultaneously. Charting improvements, greater provider adherence to established standards of care for chronic illness, and a renewed emphasis on promoting healthy behaviors during clinic visits all resulted from partnership between various providers at PCCC 4. Additional funding and promotion of the CPCI program could have improved linkages to community providers, although surveyed patients reported that the brief partnerships PCCC did establish with these entities (dietitian, mental health department) were beneficial.	Not reported
Pastore 2013 [39]	USA	Mixed methods pre/post	To determine the impact of practice enhancements on: (1) changes in missed appointments; (2) Changes in health outcomes; number of ER visits and hospitalization days; and (3) perceptions of integrated care through qualitative exit interviews with participants	PWSMI with unmet primary care needs. SMI patients were linked with an Assertive Community Treatment (ACT) team. Diagnoses included psychotic disorders, schizophrenia and schizoaffective disorder, mood disorder and bipolar disorder. Large academic family practice. The family practice site and the ACT program were in an economically disadvantaged urban setting three blocks apart.	Practice enhancements within primary care comprising a) a Behavioural Health Liaison (BHL)(RN working in the family practice) to serve as a single point of contact and as a coordinator for patients with SMI. The BHL was responsible for triaging clients, scheduling open access same day appointments, organising laboratory tests and referral where needed b) a psychiatric care basics briefing package for all practice personnel, and c) a handheld medical record known as the ‘Health Passport’.	Not reported. Study reported ER utilization and hospitalization at baseline, 12.5% went to ER for a physical health problem, 6% went to the ER for a mental health condition, 6% were hospitalized for a physical health condition; and 18.75% were hospitalized for mental health condition.	1. Continuity of the BHL who remained consistent throughout the study. This continuity was instrumental in developing trust between patients and their counsellors, and coordinating access to services with the primary care home. Patients also found that the liaison assisted with navigating through secondary referrals, coordinating specialty care, providing education, and reconciling complicated medication lists. 2. Practice liaison and ‘‘enhancements of care’’ improved navigation through the office, and culminated in a higher rate of kept appointments and therefore improved care continuity. 3. Educational sessions for primary care and ACT teams improved knowledge and understanding of the separate environments and provided assistance to PC staff to understand how to best deal and relate to SMI patients. 4. Open access card that could be given to patients—case managers could leave notes for PC on the back of the cards, and this allowed some patients to come unattended. The cards indicated to front desk staff that the patient was known to the ACT team. 5. In house radiology and pathology. 6. Same day walk in appointment and an opportunity to attend without an ACT member. 7. Access to PC enhanced access to appropriate emergent care. Behavioral health staff felt that the coordination between family practice inpatient care and discharge back into the community was greatly facilitated by care continuity.	Patient—The Health Passport was employed only by one patient. Most patients reported knowledge of the passports, but they were either lost or left at home
Perkins 2010 [40]	Australia	Mixed method evaluation	To evaluate an innovative rural service offering comprehensive primary health care for mental health service clients in a rural mid-western NSW community.	Clients of the community mental health service (CMHS) with a range of diagnoses including schizophrenia, psychotic disorders, mood disorders, substance use, personality disorders, anxiety. 20% of attendees had been clients of the Community mental health Team (CMHT) for 5 years or more and proportionately more clients with psychotic disorders relied on the GP clinic for continuing services. Single rural general practice - The GP clinic was located in an adjacent building to the CMHS service. The CMHS service was co-located with other services including a diabetic educator, women’s health and drug and alcohol services	Monthly clinic for (Serious Mental Illness (SMI) clients conducted at a single rural general practice. Appointments were arranged by a member of the CMHT who also provided transport for their client. Results of the consultations were documented in the GP and MH service records.	Not reported. The physical health status of attendees was described—52% of the clients did not have a physical health illness when assessed by the GP and most (79%) were referred to other health and community services. Physical conditions reported included vascular disease/diabetes (10%), GI disorders (7%) and other (28%)	1.The service model used only existing resources and existing funding structures so was easily incorporated into routine practice. The same GP ran the clinic providing opportunities for continuity of care, therapeutic doctor-patient relationship, consistent approach to management and GP/MH relationships and collaboration. 2. Commitment of the local PC to maintain the service and increase/decrease depending on demand. 3. Sustainability—The service was developed incrementally without external funding or formal project plan. The general practice provided a consulting room for the GP clinic each month, a GP volunteered to conduct the clinic, and the CMHT arranged for and supported clients to attend. 4. Targeted use of the clinic for clients not otherwise receiving primary health care services thereby keeping the clinic to a manageable size while maintaining relevance and reach, and preventing access block. 5. Clearer division of roles between GPs and psychiatrists.	Not reported
Rossom 2020 [41]	USA	Cluster-randomised pragmatic trial	To allow primary care clinicians and psychiatrists the opportunity to improve cardiovascular risk in a timely manner for patients with SMI.	PWSMI—defined as having bipolar disorder, schizophrenia, or schizoaffective disorder. Three healthcare delivery organisations comprising 78 primary care clinics (largely rural and geographically dispersed). The psychiatric staff were not co-located in primary care, although some behavioral health clinics were in the same building as primary care clinics (10 BH clinics but 50 + primary care clinics).	Clinical decision support (CDS) tool that provided Best Practice Alerts (BPAs) to both the primary care clinicians (PCPs) and the psychiatrist. The PCP CDS included a summary of six modifiable CV risk factors and patient-specific treatment recommendations. Psychiatrists received CDS alerts during their next visit with an eligible patient with SMI that alerted them to an elevated body mass index or recent weight gain and the presence of an obesogenic SMI medication.	Not reported	1. Secure web service linked with the EHR allowed for maximum efficiency and versatility of the CDS. 2. The CDS allowed timely identification of major CV risk factors in people with SMI and therefore opportunity to identify and intervene CV risk factor control. 3. Use of a clinical decision support (CDS) tool for intervention delivery and data collection allows for a comparison of similar data across clinics	Not reported
Scharf 2013 [42]	USA	Descriptive, mixed method evaluation	To describe the SAMHSA Primary and Behavioural Health Care Integration (PBHCI) grants program, which supports the integration of primary care services into community behavioural health settings for adults with SMI. Specifically, to describe the grantee organizations, their integrated care programs and implementation plans, and the challenges associated with this approach at start up and after one year of program implementation.	Serious mental illness (SMI)—schizophrenia, bipolar disorder, and clinical depression. 65 behavioural health agencies at 86 sites or locations. Most grantees consisted of a single treatment site, and most were in a suburban or urban setting. Less than half of the behavioural health agencies offered any primary care services prior to receiving the grant. PBHCI grantees tended to include at least one primary care provider organization, of which a majority were Federally Qualified Health Centres (FQHCs). Some primary care partners were in the same building as behavioural health providers, whereas others were up to 23 miles away (median = 1.5 miles).	Each grantee’s model of service integration included required and optional program features. The required program features were screening, assessment, and referral for the prevention and treatment of general medical illnesses and risk factors, including hypertension, obesity, smoking, and substance abuse; a registry or tracking system to house data about consumer level primary care needs and outcomes; care management, defined as individualized, person-centered planning and coordination to increase consumer participation and follow-up with primary care services; and illness prevention and wellness support services.	Not reported	Not reported	1. Recruiting and retaining staff. Other staff issues, such as conflict and low morale, were less common but more persistent, given that 3/4 continued to report these issues one year later. 2. Space for those programs that chose to bring PC providers on-site, but these were generally resolved within the first year. 3. Problems sharing consumer data across behavioural health and primary care partners for the purpose of patient care (n = 8, 14%). EHRs, registries, and data sharing challenges were present at both baseline and follow-up. Concerns with the quality and capacity of available packages. Electronic systems that efficiently and securely integrate behavioural and primary health information were not available. 4. Licensing issues, such as delays in approval for FQHCs to provide and bill for behavioural health services. 5. Approximately 1/4 grantees (n = 19, 35%) reported difficulty getting consumers to participate and keep them engaged at one year. Grantees commonly reported difficulty in getting staff to refer appropriate consumers to the program, and some reported difficulty enrolling consumers who were referred. Several reported difficulties caused by potential program participants not showing up for appointments. Thirteen grantees (24%) reported difficulties maintaining participation and following up with consumers who had already enrolled in PBHCI.

Perkins 2010 [40] and Fitzpatrick 2017 [36] reported on an Australian rural general practice clinic providing assessment and management of physical health conditions for PWSMI managed by a mental health service. A designated consultation day was set aside due to shared recognition that access to comprehensive primary care was lacking. Perkins conducted a mixed-methods evaluation of the clinic using two years of service data (2007–2009) and 15 qualitative interviews with health care staff and others associated with the clinic. Fitzpatrick used qualitative data from a different date set (16 of the interviews conducted with healthcare staff during 2015/2016).

*Hunt 2016 *[37] described a ‘boundary spanner’ role initiated in England in which two members of the community mental health team were seconded as Community Physical Health Coordinators to span the primary care and community mental health service with a focus on improving communication and collaboration. Key informant interviews, a focus group, surveys, and quantitative data were collected, and the study reported on aspects of the role itself, outcomes of the multidisciplinary meetings, and changes in reporting from GP records.

*Pastore 2013* [39] was an ‘easy-to-implement’ practice adaptation within a primary care setting in the US involving a Behavioural Health Liaison Officer, who was a registered nurse (RN) from the family practice who took on a coordination role and served as a single point of contact for patients with SMI. This study used pre/post data and a review of medical record data to evaluate the practice enhancement and use of the service.

*Rossom 2020* [41] was a randomised controlled trial (RCT) from the US that provided interim study results on the implementation of a clinical decision support (CDS) tool that alerted both primary care and mental health clinicians for a given patient with SMI about increased cardiovascular (CV) risk.

Two publications from the US were included that described early efforts to set up integrated primary and mental health services to improve the physical health care of PWSMI. The Nover pilot program 2014 [38] used a partnership model to set up a clinic serving rural low-income individuals. A series of Plan-Do-Study-Act (PDSA) cycles were used by the clinic to set initiatives resulted in new forms for referral, assessment and treatment, and a shared care plan, all for use by the treatment team. Scharf 2013 [42] described a program which supported the integration of primary care services into community behavioural health settings for adults with SMI. This publication describes the grantee organisations (agencies that were the recipients of grants), their integrated care programs and implementation plans, and the challenges associated with this approach at start-up and after one year of program implementation.

### Reporting of physical health outcomes

There was minimal reporting of physical health outcomes for patients within the included studies (Table 4). The Perkins study did not report on outcomes but described the physical health status of attendees. Over half (52%) of the clients assessed by the GP did not have a physical health illness and most (79%) were referred to other health and community services. The physical conditions reported among the participants were vascular disease/diabetes (10%), GI disorders (7%) and other (28%) [40]. In the Hunt study, although no physical health outcomes were reported, the study highlights that as a result of the multidisciplinary team meetings and audit of GP systems, disease reviews and actions related to physical health checks, and medication reviews were the most frequently discussed issues. The study reports improvement in recording of CV risk and improvement in the recording of systolic BP, HDL, smoking status, and BMI which was reported to improve from 38% of service users (*n* = 115) to 58% (*n* = 79) of users post project [37]. The Pastore study also did not report physical health outcomes but reported that at baseline 12.5% of patients went to the ER for a physical health problem, and 6% were hospitalised for a physical health condition [39].

### Contribution of the literature to the research question.

To meet our definition of shared care, all included studies involved a general practice or equivalent. The studies reported a range of outcomes including feasibility, service operation and acceptability, provider viewpoint, provider reporting mechanisms and quality of life (Table 4). This review however was interested in the barriers and facilitators to the engagement of primary care in shared care for people with SMI. Therefore, to address our specific research question, we have separated our results as such. The barriers and facilitators reported tended to refer to the program/intervention as a whole rather than by individual services or health professionals. Therefore, while the discussion that follows is relevant to primary care and GPs, it may also be relevant to other health professionals involved in shared-care arrangements.

### Facilitators to the participation and engagement of primary care in shared-care arrangements with community mental health services for preventive care of PWSMI

Although the yield from the database search was modest, we identified some items from the literature that had the potential to enable or enhance the participation and engagement of primary care in shared-care arrangements around the physical health needs of PWSMI. These included:

- Service models

Perkins [40] and Fitzpatrick [36] reported on the establishment of a GP clinic for consumers of the local mental health service in a small rural town of NSW with about 8,000 residents, in which designated appointments were set aside on a specified day to see patients with SMI. The authors report that there was little deviation from usual practice and no requirement to invest in new systems or to change service systems or the way staff work. While this clinic aimed to improve access to a GP rather than shared care, this model could also provide an opportunity for shared care to be established because of the increased collaboration between the practice and the mental health service based around individual clients. The authors argued that the key to success was the simplicity of the model; inherently no requirement by GPs to drastically change the way they practiced, no additional cost to services and a perceived mutual benefit by the partners involved [40]. They also acknowledged that a similar model might not be generalisable to other settings and was reliant on the ‘nimbleness, flexibility, resourcefulness, and persistence’ of individual health professionals to both set up and maintain the clinic [36].

b) Specific roles to promote communication and coordination within existing systems

Most studies incorporated a specific role designed to provide active coordination [36–40]. These roles were not specifically based in general practice, and the outcomes assessed when these roles were utilised, tended to be how the role enhanced working relationships or cross service processes, or impacted patient outcomes. Generally speaking, coordination roles were seen as valuable because they improve the quality of care for patients [36]. When specifically designed to promote sharing or collaboration around certain tasks, the clinical and coordination ambiguity is removed and accountability for actions applied to specific individuals [39], resulting in better patient monitoring and subsequently better outcomes. Within this review we identified a variety of roles built into larger programs or operating as standalone interventions including Community and Physical Health Co-ordinators [37], Behavioural Health Liaison officers [39] and nurse-led coordinators [38]. Although these roles varied in name and function, at their core, they aimed to generate greater engagement in patient care and participation by all services. Engagement was enhanced because the roles fostered trust, created a link between services and improved respect for professional roles across the services [37]. At their simplest, the roles allocated a designated person to make and monitor appointments and transport people to appointments [36, 40]. The more complex arrangements used various staff who, through a variety of functions, could ‘span the boundaries’ between the primary care and mental health service [37, 39]. Both existing staff members [37, 40] and new staff [38] were allocated to take on these roles. Some shared arrangements utilised a prior position with designated liaison tasks with, or knowledge of, the collaborating services [36, 37, 39, 40]. These therefore drew on existing working relationships to promote effective mediation or liaison between the services.

While not a specific focus of this review, better engagement of patients was also associated with these roles because they incorporated a navigation function through secondary referrals and specialty care [39]. Coordinators also provided patient education to reconcile complicated medication lists, resulting in a higher rate of kept appointments and therefore improved care continuity [39]. In the study by Nover [38] for instance, nurse-led care coordination for medication support, illness management and referral/follow up was the most frequently requested service by patients.

c) Improved collaboration and teamwork

In the Nover [38] study outcomes in relation to teamwork were more notable than the service quality and patient outcomes. This study reported that the psychiatric and primary care providers worked more closely together on patient health outcomes, that there was greater provider adherence to established standards of care, and a renewed emphasis on promoting healthy behaviours as a result of the partnership [38]. Providing better outcomes for patients is the core business of all health-related services, therefore achieving measurable benefits for patients engages health providers in the process because they can see the results that this brings. The fostering of clear roles and expectations creates a working environment where collaboration can be mutually beneficial, and there are realistic aims and expectations [36].

Joint care planning is acknowledged to promote engagement and partnership of health services around the needs of individual patients without making dramatic structural change [14]. In the Hunt study [37] the multidisciplinary team (MDT) meetings provided an avenue for multiple perspectives to be incorporated into an effective management plan for patients. Identifying key objectives and actions related to these allowed the team to ‘co-evolve’, develop new ways of communicating and working together, and to share and integrate knowledge to improve the physical health care of service users [37]. When shared care planning could be achieved more efficiently through the use of decision support systems, shared Electronic Health Records (EHRs), or other electronic means, there was greater provider adherence to established standards of care for chronic illness, and a renewed emphasis on promoting healthy behaviours [38]. Identifying major risk factors in PWSMI improved the opportunity for health professionals to intervene and to improve risk factor control in a timely way [41].

Enhancing communication between service providers, whether it be through MDT meetings, boundary roles or system changes acts to increase the willingness to collaborate [37]. Increased communication allows each provider to better understand both the medical and psychiatric needs of the patient [39]. This was identified as a key facilitator for designing and delivering appropriate management plans because it provided an opportunity to discuss individual cases, secure clinical consultation or specialist advice, provided new avenues for professional learning and opportunities for additional skill development [37, 39].

### Barriers to the participation and engagement of primary care in shared-care arrangements with community mental health services for preventive care of PWSMI

The literature provided some indication of specific issues or factors that appeared to create a barrier or blockage to the participation/engagement of health services and health professionals in shared-care arrangements around the physical health needs of PWSMI.

- Willingness, confidence, and capacity

Shared care will only be successful if services and individuals have willingness to participate, confidence to carry out the required tasks and access to the physical structures that produce capacity (e.g., staffing, space). Experiencing problems in these areas will automatically impact service and individual engagement as it increases the time required to participate, the level of frustration experienced and ultimately impacts the perceived value of the activity.

The commitment and willingness of individuals was a resounding factor in the Perkins and Fitzpatrick studies [36, 40]. The idea to start a GP clinic for SMI was a joint decision by an individual GP and the community mental health service. In effect this GP volunteered his/her practice and time to provide the clinic, although he/she was still able to be remunerated through Medicare. Fitzpatrick [36] also noted widespread concerns by GPs about their ability to care for clients with complex SMI pointing to a perceived lack of experience, confidence, or knowledge. Providing education to staff involved in shared-care arrangements was offered to help them engage in the process and to feel confident in participating [38, 39]. This was deemed necessary for primary care staff so they could understand the behaviours they might experience and be able to respond accordingly [39], to increase the understanding around the importance of treating mental and physical health simultaneously [38] and also to increase the knowledge and understanding about the separate environments in which all partners work [39].

Larger projects in the US that reported on early integration across multiple sites also reported experiencing challenges related to staffing, organisational resistance, and internal capacity issues [42]. These included recruiting and retaining staff, general turnover and having sufficient space to provide an integrated service [38, 42]. Being able to adequately provide recovery-oriented mental health care also presented barriers and frustration among service providers. Within one Australian study, the limited capacity of the clinic to collaborate with other community and social services was noted [36]. Despite incorporating key aspects of recovery-oriented practice such as advocacy, social inclusion, and the support of individuals and their families to self-manage their physical and mental health, the study found less evidence for the necessary infrastructure and supportive relationships required to strengthen the recovery orientation of service delivery to include housing, employment, and income and vocational training support, all of which are recognised key elements to achieving positive recovery outcomes.

b) Funding and incentives

Overwhelmingly, the need for adequate funding, funding restructure and monetary incentives to appropriately support shared care came through in this review. Poor alignment of incentives and payments with patient needs were seen as reinforcing previously established practices which effectively siloed services and excluded PWSMI [36]. This study (Fitzpatrick 2017) also noted that the GP clinic appeared constantly ‘under siege’ by the political and economic understandings that determined what should be considered as cost-effective and efficient practice. For example, having multiple people in a consultation for one person can be perceived as time wasting or inefficient from a payment/funding standpoint.

Delays in payments for services through bureaucratic processes [42] and missed provider payments if patients with SMI failed to turn up for appointments [36] all proved problematic in this review. General practice in Australia runs on a fee for service/small business model in which funding delays or inadequate funding may impact their engagement in shared care. Even programs with secured funding for the integration of primary care and mental health, noted a deficiency in adequate resources to promote the program more widely in the community and among allied services such as dieticians [38].

c) Inability to share information

A common stumbling block for shared-care arrangements is the inability to share information about patients between services due to incompatible IT systems, or approval to access these systems. The inability to access and update information in real time adds pressure to work practices and frequently introduces repetition, particularly if information must be updated manually, is in multiple places, or is not accurate or up to date when it is required [37]. The Rossom study tested a clinical decision support system that could link with the EHR and provide alerts for all treating parties as a way of negotiating these split systems. The study found that this resulted in reduced disruption, and real time updates resulted in increased accuracy [41].

Across multiple sites within the Scharf study, EHRs, registries, and data sharing challenges were present at both baseline and follow-up. Although some software options were available to be bought that might provide a workaround, there were serious concerns from services regarding the quality and capacity of available packages, and electronic systems that efficiently and securely integrated behavioural and primary health information were simply not available [42].

d) Challenges in engagement with PWSMI

PWSMI are a hard-to-reach group, and this makes it challenging for health services to provide the required care even with good, shared care systems in place [37]. This has traditionally caused problems with engagement of patients [37, 40] and providers alike since there are many points at which care can be derailed. The need for frequent in-patient care can further disrupt, particularly if the persons social structures (family/carers) and housing are impacted.

Within the Scharf study approximately one in four grantee organisations providing integrated care for PWSMI reported difficulty recruiting and retaining consumers. They reported (Page 663) “Many clients [are] referred to the program, but then they will either avoid our case manager’s attempts to contact them, or they will not show up for their intake” [42]. This study recommended the development of care managers and the use of peer specialists who may have the time and skills to engage with clients and ensure they attend appointments [42]. Hunt [37] suggested that more work be done to address access for difficult to reach groups. Studies that used tools to aid engagement of patients like health passports and support groups found they were poorly utilized [37, 39].

## Discussion

Seven studies met our criteria for this review. We identified variable contribution to the research question of barriers and facilitators to the engagement of GPs in shared-care arrangements for PWSMI from this literature. This may relate in part to the parameters used within the review, and a broader focus on integrated services within the literature, as opposed to studies where GPs and the mental health service share responsibilities for care but otherwise operate as separate entities, as within the Australian context. This suggests the need for more research which evaluates GP participation or engagement more fully, particularly around the physical health needs/preventive care for PWSMI when primary and specialist mental health services are equally involved in patient care.

The studies identified used a variety of strategies to support shared care including special clinic sessions, boundary-spanner workers, decision-support tools, multidisciplinary shared-care plans, and shared health records. There was limited reporting of patient physical health outcomes in the studies as a result of these strategies. In some cases, studies highlighted that the strategy brought more focus to assessing physical health issues for PWSMI, or that clinical documentation or risk factor reporting improved. This did not translate to obvious improvements in physical health. Key facilitators of engagement included having a good fit with current organisation and practice, providing a dedicated role to coordinate the parties and help patients to navigate appointments, utilising multidisciplinary teams and facilitating shared health records in real time. Barriers included lack of motivation and confidence of primary care practitioners, poor alignment of funding/incentives, inability to share patient information and other demands on the life and care of PWSMI.

These findings are broadly consistent with other general research about shared care between specialist and primary care for people with long-term conditions. A recent review on collaborative care for depression found several barriers including lack of commitment, limited resources, poorly integrated information and communication systems, poor coordination of finances and care pathways and conflicting objectives. This review also stressed the importance of agreeing on guidelines for care, roles and responsibilities and “willingness to co-work and co-learn” [43]. A review of GP engagement with specialist palliative care services identified the importance of communication and information sharing, clear roles and pathways for referral and time and capacity within primary care [44]. An overview of reviews of interprofessional collaboration in primary care settings identified lack of time and training, lack of clear roles, fears relating to professional identity and poor communication as the main barriers [45]. Facilitators included co-location and a shared understanding of roles.

A feature of shared-care for PWSMI, that is not always present for shared-care with other populations, is the level of difficulty engaging consumers in the process. This was reported as a major recruitment and retention issue within the Scharf study [42] and Hunt reported that despite improvements in knowledge sharing among health care providers, access and engagement remained a problem for some ‘difficult to reach’ users [37]. There is a significant body of research on how PWSMI do not access primary care for preventive care for a range of reasons including patient factors (e.g., cognitive challenges, psychopathology) and provider factors (e.g., provider bias, skills and confidence in working with PWSMI) [8, 10]. The difficulties with shared care described above, sit on top of the difficulties in engaging PWSMI in primary care in the first place.

**Fig. 2:**
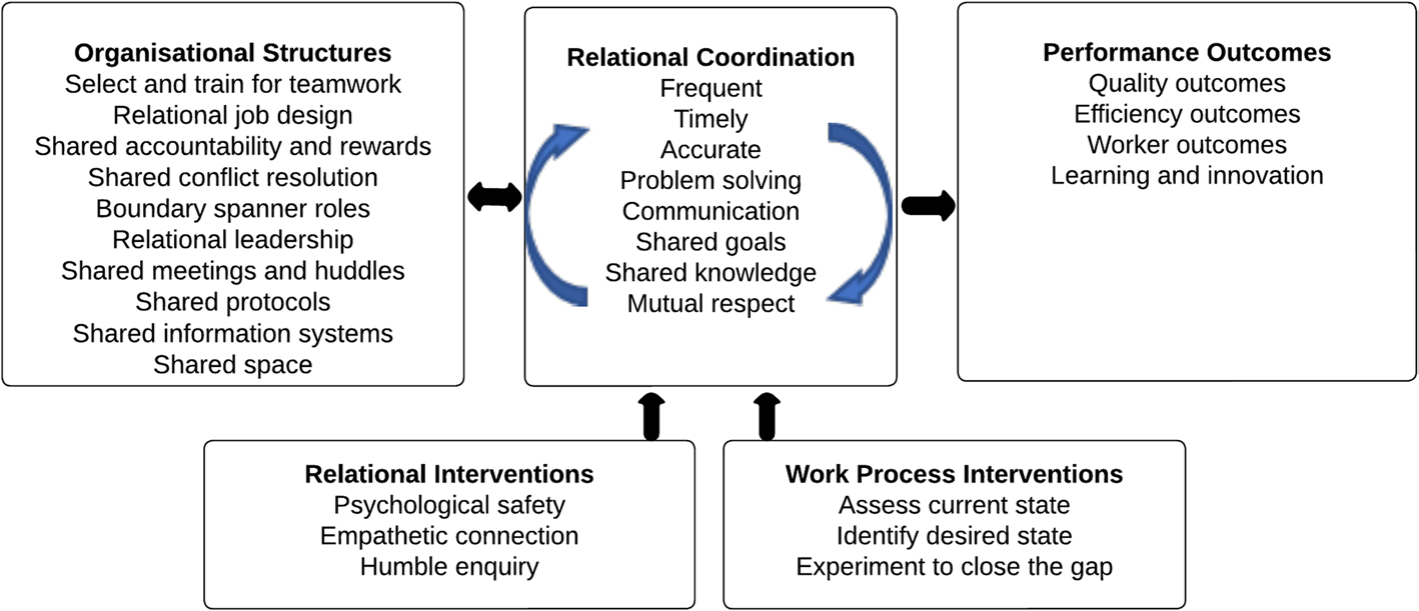
Relational Coordination Theory. Based on the figure from Bolton et al. 2021. Revisiting Relational Coordination: A Systematic Review. Journal of Applied behavioural Science 57:002188632199159. Permission to use this figure was obtained from Sage Publishing 23/01/2023

The review findings broadly align with Relational Coordination Theory. This framework assesses teamwork, in which having shared goals, shared knowledge, and mutual respect is seen to foster timely, accurate, problem-solving communication, enabling stakeholders to effectively coordinate their work across boundaries. These attributes are mutually reinforcing and are underpinned and supported by cross-cutting organisational structures and processes that encourage and increase stakeholder collaboration (Fig. 2). The theory further contends that strong networks of relational coordination facilitate the achievement of quality, efficiency, worker and learning outcomes [46, 47].

Within this review there was evidence to support a number of relational components (Table 5) particularly shared goals, willingness to participate, increased communication and shared knowledge. Communication and relationships among healthcare professionals are particularly important when services operate as separate entities and essential if they are to improve the care of the patients they share. Within this review there was a focus on providing education to health care providers, not only to improve knowledge, confidence, and capability but also to improve their understanding about the services that they work closely with. Having shared accountability is particularly important in shared care arrangements to streamline the work and also to ensure that individual tasks are achieved. When services work together and experience these benefits, this acts as a mutual reward, which also comes from knowing patients are receiving appropriate care and continuity in their care. Organisationally, services benefited from ‘boundary spanner’, ‘hinge’ and case-coordination roles that reach out into primary care, having shared multidisciplinary meetings and communication systems and being able to share medical information in a timely way. Having guidelines and standards of care that were mutually agreeable and relevant was also desirable.

**Table 5:** Relational coordination theory domains identified within the included studies

**Study**	**Organisational Structures** (Cross cutting structures that support relational coordination)	**Relational Coordination** (Mutually reinforcing process of communicating and relating for the purpose of task Fig. 1. From a linear to dynamic theory of relational coordination)	**Outcomes** relevant to relational coordination (Quality, Efficiency, Worker and Learning)
Fitzpatrick 2017 [36]	• 'Hinge person' who takes on the responsibility with the GP to manage bookings and client organisation • Designated monthly clinic • Basic infrastructure, structural support (administrative, financial, and technological resources) • Co-location or proximity of providers • Leadership and management Skill development e.g., management of clozapine/prescribing of other medications	• Shared rewards – seeing patients get better • Opportunities for the development of professional team culture, stability, and collaborative working relationships (influenced by rural location). The importance of developing close working relationships was considered critical in order to establish realistic aims and expectations	Reduction in referrals from the GP to the mental health team when prescribing can be done without the need for additional referrals
Hunt 2016 [37]	• Boundary-spanner role—help to “facilitate transactions and the flow of information between people or groups who either have no physical or cognitive access to one another, or alternatively, who have no basis on which to trust each other” • Shared multi-disciplinary meetings and a method for tracking actions • Role legitimacy and commitment providing an official mandate to perform their responsibilities. By appointing an individual to this role, rather than simply implementing a set of guidelines and protocols, a process was developed whereby changes and improvements were designed and embedded into practice • Community and primary care staff worked together to develop a flowchart of responsibilities for the CPHC role which helped to provide some consistency and continuity across different teams Shared paper-based Care Planning Approach (CPA) document between the services	• Information sharing between the GP practices and CMHT, which helped to improve working relationships between services and promoted a greater understanding and respect for each other’s professional roles • MDTMs gathered multiple perspectives and shared information across services enabling the teams to provide a coordinated approach to the care of service users • The MDT meetings provided primary care and community staff with a space to share and acquire knowledge concerning service users’ physical and mental health in a supportive environment • Shared Knowledge/knowledge transfer—"learning by meeting"- The MDT meetings provided an opportunity for staff to understand the relationship between mental and physical health; understanding how physical health impacts on mental health and vice versa • Knowledge integration was focused on combining service user data and information from multiple perspectives around key objectives with an action orientated focus, rather than simply sharing information in a passive way. As this was a new process the team were able to co-evolve and develop new ways of working and new processes to communicate, share and integrate knowledge to improve the physical health care of service users	The introduction of the boundary spanner role and multidisciplinary meetings improved the management of physical health care for people with SMI, particularly through sharing of information, co-ordination of actions, and proactive delivery of care via joint action plans for the physical health management of service users which are discussed and appraised at multidisciplinary meetings
Nover 2014 [38]	• Designated nurse and social worker positions/roles PDSA changes to systems to promote better assessment of patients and better collaboration	• Improved interagency teamwork	Quality improvement and treatment were stated to be the outcomes of interest, but no rigorous processes were put in place to formally measure these Study states "Charting improvements, greater provider adherence to established standards of care for chronic illness, and a renewed emphasis on promoting healthy behaviours during clinic visits all resulted from partnership between various providers Expected outcomes include decrease in health disparities, higher QoL, less disparity and less stigma
Pastore 2013 [39]	• Behavioural health liaison role selected from the registered nurses at the family practice site to assist with access and coordination of care • Primary care home model A Psychiatric Care Basics Tool Kit and training for staff	Not discussed	There was evidence to suggest that the number of missed appointments significantly decreased with the addition of the practice enhancements (from 42 to 11). This difference was statistically significant (p < .01) No changes in health outcomes, ER utilisation or hospitalisation Enhanced access to appropriate emergent care
Perkins 2010 [40]	• Focus on utilising existing structures and funding streams • The clinic was developed incrementally and with NO formal project plan • ‘Solid agreement underpinning the service'	Publication states "There is a good working relationship between the surgery and community mental health team. Replacing the current clinic GP if he should ever leave is not a general concern because of the commitment of the local surgery to maintain the service”	Improved access and continuity of GP care—Anecdotally the clinic improved access to primary care for mental health clients which was supported by attendance between 38 and 54 individuals (19–27% of all CMHT clients) accessed the GP Clinic each 6 months. and repeat attendance at the clinic (40% of clients) Reduced attendance at the MH inpatient unit -Perceived reduction. Not formally assessed
Rossom 2020 [41]	• Shared stakeholder planning—healthcare systems leaders from all care systems representing both primary and mental health specialty care developed consensus regarding content and workflow of the intervention • Training sessions for PCPs and rooming staff occurred via in-person presentations or online training, reviewed written training material, received training directly from clinic leaders or attended a live presentation • Hosting the CDS on a secure web service securely linked with the EHR allowed for maximum efficiency and versatility • Best practice advisory (BPA) alerting clinicians to patients with SMI with at least one modifiable CV risk factor not at goal. Identification, alert to psychiatric prescriber and intervention for patients with SMI who were on an obesogenic medication for SMI and had either a BMI > 25 or had gained 7% or more of their body weight in the previous year	Not discussed	Not reported
Scharf 2013 [42]	• Programs differed in their structures. Most grantees (*N* = 34, 61%) reported that they had or were developing electronic health records (EHRs) for behavioural health information, and 46% (*N* = 26) reported using or planning to use EHRs for general medical data. Plans for shared general medical and behavioural health EHRs were less common (*N* = 16, 29%) • Care manager roles recruited from additional money to recruit staff • No shared protocols—implementation challenges related to merging primary care and behavioural health protocols and barriers related to billing and administrative issues were reported	7% of grantees described staff conflict related to the program and lack of staff buy-in or low morale	Not reported

This review provides some future considerations for the design of shared care programs between mental health services and GPs providing preventive care for PWSMI. The traditional focus on education and guidelines, while useful, is likely to be insufficient to achieve shared care even for programs that involve minimal changes to general practice. Joint development and co-design of evidenced-based guidelines that have the input and support of both general practice and mental health and which clearly delineate responsibilities [48] may be beneficial however, effective shared care requires considerable changes to systems and structures and achieving these changes and establishing new systems can take considerable time. The ability to share patient information that is accurate, updated in real time and readily accessible by all parties within health information systems seems crucial and is currently a major barrier [49]. Measures to improve the connectivity of these systems with shared or accessible clinical records without adding additional processes that take extra time or lead to treatment burden for patients [49] would be highly valuable. Hence, commitment to providing appropriate and sustainable funding, incentives and support would be required across the participating services. Additional roles that provide coordination and promote collaboration such as boundary spanners and care coordinators can be valuable because they promote the working interface between services, they take on those tasks that often cannot be routinely achieved in clinical settings, and they develop the structures that encourage individual services to better communicate and work together. Therefore, providing nurse navigator and peer worker roles may assist PWSMI to navigate the complexity and also help provide coordination between services.

In any shared arrangement, contextual factors also need to be considered such as the stressors operating on the workforce. This implies that shared care relationships need to be underpinned by policy to provide sufficient capacity within general practice systems to constructively engage with shared care. This could include MBS funding for health assessments, QI activities or case conferences, and additional roles such as nurse navigators [50].

The positive aspects of the Relational Coordination model (shared goals, willingness to participate, increased communication and shared knowledge), although ideal, may not be easy to achieve in practice. Services are not static entities and the cultural and organisational patterns within services are often deeply embedded. This has considerable implications for practice. As indicated in the model, some experimentation to ‘close the gap’ may be required. Achieving better working relationships across services may need to happen slowly, through testing of strategies to see what works locally, and by making relevant and incremental changes. More research would be required however to identify the optimal way to achieve this in practice.

### Strengths and limitations

The strength of this review lies in the systematic approach used to identify relevant studies and the use of recognised reporting frameworks. This review was limited by the lack of research specifically dedicated to answer the question of how to improve the engagement of primary care and general practitioners in shared care with PWSMI and their mental health services. A broader focus on integrated services within the literature may have resulted in missed opportunities to explore different collaborations, however this review was specifically looking at separate services and how they might work better in a shared arrangement. There was also limited reporting of physical health outcomes as a result of the interventions, therefore we cannot draw any conclusions about the evidence for changes in health outcomes resulting from changes to shared care arrangements. Study evaluations were often conducted over relatively short time frames, and this may have been insufficient time to identify system changes, as this can take considerable time. Research was not identified to provide evidence-based guidance on improving engagement in shared care in diverse settings (e.g., rural settings). As such any recommendations made within this review are not generalisable.

## Conclusion

Consistency of these results with other research on shared care between specialists and primary care for people with other long-term conditions (e.g., cancer) suggests much of the broader literature on shared care is likely to be applicable to the context of GP-MHS shared care. However, PWSMI present particular challenges for recruitment and retention to a shared-care program. Our findings were consistent with relational coordination theory, which posits that performance outcomes rely upon providers sharing “goals and knowledge, mutual respect” and engaging in “frequent, timely, accurate, problem-solving communication”, supported by a range of structures such as shared information systems and roles that span services. These factors may be more important in engaging primary care in shared care arrangements than the traditional focus on incentives, education, and guidelines.

### Supplementary Information

**Additional file 1.**

## Data Availability

All necessary data supporting the conclusions of this article are included within the article and associated files. The protocol for this scoping review was made available prior to the conduct of the review in Open Science Framework (https://osf.io/?view_only=3b6464270fa7496c90a5cf3b2930302a).

## Abbreviations

PWSMI: People with severe/serious mental illness
SMI: Severe/serious mental illness
GP: General practitioner
MHS: Mental health services
MeSH: Medical subject heading
OECD: Organisation for Economic Cooperation and Development
PRISMA-ScR: Prisma extension for scoping reviews
RN: Registered nurse
RCT: Randomised control trial
CDS: Clinical decision support
CV: Cardiovascular
PDSA: Plan-Do-Study-Act
EHR: Electronic health record
